# Postoperative Coagulation Changes in Patients after Epicardial Left Atrial Appendage Occlusion Varies Based on the Left Atrial Appendage Size

**DOI:** 10.3390/diseases12010008

**Published:** 2023-12-29

**Authors:** Jakub Batko, Jakub Rusinek, Artur Słomka, Radosław Litwinowicz, Marian Burysz, Magdalena Bartuś, Dhanunjaya R. Lakkireddy, Randall J. Lee, Joanna Natorska, Michał Ząbczyk, Bogusław Kapelak, Krzysztof Bartuś

**Affiliations:** 1 CAROL—Cardiothoracic Anatomy Research Operative Lab, Department of Cardiovascular Surgery and Transplantology, Institute of Cardiology, Jagiellonian University Medical College, 31-008 Krakow, Poland; 2 Thoracic Research Centre, Collegium Medicum Nicolaus Copernicus University, Innovative Medical Forum, 85-094 Bydgoszcz, Poland; artur.slomka@cm.umk.pl; 3 Department of Pathophysiology, Nicolaus Copernicus University in Toruń, Ludwik Rydygier Collegium Medicum in Bydgoszcz, 85-094 Bydgoszcz, Poland; 4 Department of Cardiac Surgery, Regional Specialist Hospital, 86-300 Grudziądz, Poland; 5 Department of Pharmacology, Jagiellonian University Medical College, 31-008 Krakow, Poland; 6 The Kansas City Heart Rhythm Institution and Research Foundation, HCA MIDWEST HEALTH, Second Floor, 5100 W 110th St, Overland Park, KS 66211, USA; 7 Department of Medicine and Cardiovascular Research Institute, University of California, San Francisco, CA 94158, USA; 8 Institute of Cardiology, Jagiellonian University Medical College, 31-008 Krakow, Poland; jnatorska@szpitaljp2.krakow.pl (J.N.); michalzabczyk@op.pl (M.Z.); 9 Department of Cardiovascular Surgery and Transplantology, Institute of Cardiology, Jagiellonian University Medical College, 31-008 Krakow, Poland

**Keywords:** left atrial appendage, atrial fibrillation, left atrial appendage occlusion, LARIAT, coagulation, thrombolysis, left atrial appendage anatomy, coagulation factors

## Abstract

Left atrial appendage occlusion affects systemic coagulation parameters, leading to additional patient-related benefits. The aim of this study was to investigate the differences in coagulation factor changes 6 months after epicardial left atrial appendage occlusion in patients with different LAA morphometries. This is the first study to analyze these relationships in detail. A prospective study of 22 consecutive patients was performed. Plasminogen, fibrinogen, tPA concentration, PAI-1, TAFI and computed tomography angiograms were performed. Patients were divided into subgroups based on left atrial appendage body and orifice diameter enlargement. The results of blood tests at baseline and six-month follow-up were compared. In a population with normal LAA body size and normal orifice diameter size, a significant decrease in analyzed clotting factors was observed between baseline and follow-up for all parameters except plasminogen. A significant decrease between baseline and follow-up was observed with enlarged LAA body size in all parameters except TAFI, in which it was insignificant and plasminogen, in which a significant increase was observed. Occlusion of the left atrial appendage is beneficial for systemic coagulation. Patients with a small LAA may benefit more from LAA closure in terms of stabilizing their coagulation factors associated with potential thromboembolic events in the future.

## 1. Introduction

### 1.1. Atrial Fibrillation Epidemiology, Complications and Treatment

Atrial fibrillation (AF) is the most common cardiac arrhythmia, with an estimated lifetime risk of one in three patients [1]. It is estimated that approximately 15% of patients with AF are undiagnosed [2]. AF is associated with serious complications, including impaired quality of life, stroke and death [1,2]. To assess the risk of stroke in patients with AF, the CHA2DS2-VASc score is used to determine whether antithrombotic therapy should be initiated [3]. In addition, the HAS-BLED score should be evaluated to determine whether the patient is at an increased risk of bleeding [4]. Standard treatment for stroke prevention includes oral anticoagulation, with nonvitamin K antagonist oral anticoagulants being preferable to vitamin K antagonists [5]. As an alternative to lifelong anticoagulant therapy, left atrial appendage occlusion (LAAO) can be performed to eliminate the main source of thrombus in the human body—the left atrial appendage (LAA) [6]. In the past, several procoagulant factors were defined as being associated with thrombus formation in the left atrial appendage [7,8].

### 1.2. The Left Atrial Appendage Occlusion Procedures

Closure of the left atrial appendage can be performed in several ways, including percutaneous endocardial access (Watchman, Amplatzer) and epicardial access (Lariat [SentreHEART Inc., Redwood, CA, USA, now AtriCure Inc., USA], AtriClip [AtriCure Inc., Mason, OH, USA) [9,10,11]. It is worth noting that each method has device-specific limitations, such as the size of the LAA orifice or the anatomic shape of the LAA body, which may be associated with an increased risk of complications in certain cases [12,13].

### 1.3. The Left Atrial Appendage Systemic Impact

The nonthrombogenic role of LAA is still under investigation. There is still a lack of knowledge about the mechanisms associated with decreased, however, still present risk of thrombotic complications, including device-related thrombus, observed after this procedure [13,14,15,16]. To date, the neuroendocrine, hemodynamic, arrhythmogenic, and regenerative roles of LAA have been described, although the effects of LAAO on these features are not fully understood [17,18,19,20,21]. Recently, it was discovered that LAAO can influence not only stroke risk but also systemic coagulation parameters, leading to additional patient-related benefits [22]. However, it is not yet known whether patient-specific LAA anatomy can be used as a predictor of better postoperative systemic outcomes.

### 1.4. The Aim of the Study

In this study, we focused on well-established laboratory parameters that assess both blood coagulation and fibrinolysis. These markers not only reflect hypercoagulable states but are also involved in modulating various stages of hemostasis; hence, they may help assess not only the risk of thrombus formation but also in understanding the pathophysiology of blood coagulation and fibrinolytic disorders in patients undergoing the procedure of left atrial appendage closure.

The aim of this study was to investigate the differences in coagulation factor changes at 6 months in patients with epicardial left atrial appendage occlusion and different LAA morphometries. This is the first study to analyze these relationships in detail.

## 2. Materials and Methods

### 2.1. Characteristics of the Patients

A prospective study of 22 consecutive patients was performed. All eligible patients had electrocardiographically confirmed long-term AF and were free of LAA thrombi on transesophageal echocardiography. These patients were at high risk of stroke and bleeding from continued long-term systemic anticoagulation. The detailed patient characteristics can be found in Appendix A, Table A1.

### 2.2. Laboratory Tests

Laboratory investigations, blood sampling, laboratory tests, and calibrated automated thrombogram protocol were performed in accordance with a protocol published in our previous study [22]. Blood samples were collected from an antecubital vein in vacutainer tubes (containing 0.109 M sodium citrate and CTAD (buffered citrate, theophylline, adenosine and dipyridamole)). In all cases, before the procedure, anti-Xa activity (IU/mL) was measured. Tissue plasminogen activator (tPA) antigen, PAI-1 antigen, TAFI antigen and plasminogen activity were determined by ELISA (Hyphen BioMed, Neuville-Sur-Oise, France). Additionally, TAFI functional analysis was performed using retardation of clot lysis by TAFI antigen. The fibrinogen level was measured in terms of the fibrin polymerization function using the Clauss method. Plasminogen was reported as its percentage activity, while TAFI was measured as having a potency of 100%.

Patients were hospitalized for 1 or 2 days before the procedure. Patients receiving long-term vitamin K antagonists (VKAs) or direct oral anticoagulants (DOACs) were eligible if their anticoagulation was stable within the last 3 months. Prior to the procedure, bridging therapy with low-molecular-weight heparin was administered. VKA was discontinued before five days, and DOAC was discontinued at least two days before the LAAO procedure. In patients receiving aspirin or no oral anticoagulants, low-molecular-weight heparin was started at least 2 days before the procedure. The last dose of low-molecular-weight heparin was administered > 12 h before the procedure.

### 2.3. Left Atrial Appendage Occlusion Procedure

All LAAO were performed with a Lariat device (SentreHEART Inc., Redwood, CA, USA, now AtriCure Inc., USA). Postoperative transesophageal echocardiography was performed to ensure no postprocedural leakage. This method was described in detail in our previous study [23]. On the first postoperative day, all patients were continued on low-molecular-weight heparin until hospital discharge. All patients were discharged with aspirin monotherapy (150 mg/day).

### 2.4. Image Processing and Analysis

**Figure 1:**
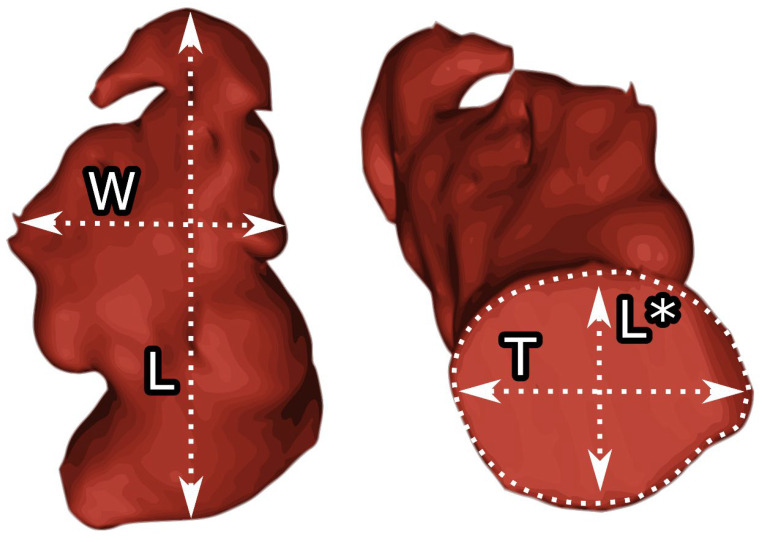
Measurements of the left atrial appendage. W—body width, L—body length, T—the left atrial appendage orifice transverse diameter, L*—the left atrial appendage orifice longitudinal diameter.

Contrast-enhanced electrocardiogram-guided computed tomography angiograms from 22 patients aged 66.2 ± 8.7 years, including 59.1% women, who were eligible for LAAO, were analyzed. The patients with heart rates greater than 70 bpm were administered 10 or 40 mg of propranolol or 40 mg of verapamil before the procedure on the recommendation of the physician. Imaging parameters for the CT were: 350–400 mA effective tube current and 100–120 kV tube voltage. The collimation was 2 × 32 × 0.6 mm, and the temporal resolution was 165 ms. During the imaging procedure, an iodine-containing contrast agent (Visipaque, GE Healthcare, Chicago, IL, USA) was injected at a dose of 1.0 mL/kg at a rate of 5 mL/s. For the test bolus, the acquisition delay was the time of maximal density of the ascending aorta with an additional 6 s. Images were reconstructed using the B26f and B46f kernels and a 512 × 512-pixel image matrix. The 70% phase of the multiphase reconstruction (10 to 100%) was assessed as the left ventricular end-diastolic (ED) phase and further investigated. The left atrium and LAA in the end-diastolic phase were segmented and processed semiautomatically using Mimics Innovation Suite 23.0 visualization and three-dimensional reconstruction software (Materialise, Plymouth, MI, USA). The LAA type was evaluated. The area of the LAA ostium, transverse length, and longitudinal length were measured. The angle of the LAA body was measured. The width and length of the LAA body were measured, and the LAA body index was calculated by multiplying the width and length (see Figure 1).

### 2.5. Statistical Analysis

Patients were divided into two subgroups: Patients with an LAA body index above average (cutoff defined as 1000 mm^2^) were included in the enlarged LAA body group, and patients who did not meet this requirement were included in the small LAA body group. In addition, patients with a larger than average LAA orifice area (cutoff defined as 23.6 mm in longitudinal orifice dimension), as proposed in previous research, were classified as enlarged LAA orifice patients, whereas patients with a smaller than average LAA orifice area were included in the small LAA orifice group [24]. Collected data were tested for normality using the Shapiro–Wilk test, tabulated and reported as means with standard deviations [mean ± SD]. The U-Mann–Whitney test was used to compare LAA morphometric characteristics and coagulation factor levels in blood samples between subgroups in the previously described populations. The Wilcoxon test was used to analyze the differences between the results of the blood samples at baseline and after 6 months in each subgroup separately. Results were considered statistically significant when the *p*-value was less than 0.05.

## 3. Results

Detailed general characteristics of the LAA are provided in Appendix A, Table A2.

### 3.1. LAA Body Size Coagulation Impact

**Figure 2:**
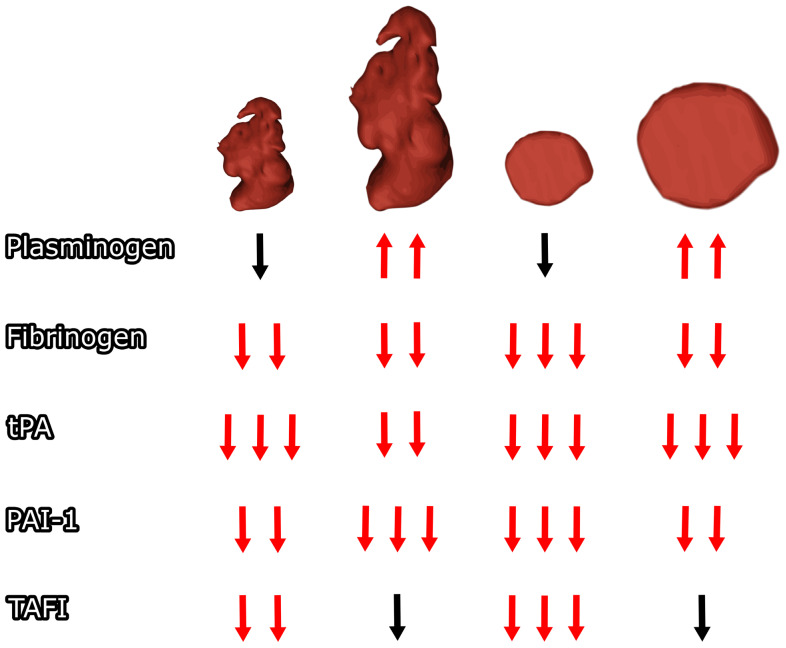
The left atrial appendage orifice and body size impact on postoperative coagulation changes. Arrows for statistically significant (*p* < 0.05) different results compared to baseline are marked red. Different numbers of arrows represent significant differences between subgroups in follow-up. tPA—tissue plasminogen activator antigen, PAI-1—plasminogen activator inhibitor type 1 antigen, TAFI—thrombin activatable fibrinolysis inhibitor.

A significant decrease in analyzed coagulation factors between baseline and follow-up was observed in normal LAA body size for all parameters except plasminogen, for which a statistically nonsignificant decrease was observed. A significant decrease between baseline and follow-up was observed in enlarged LAA body size for all parameters except TAFI, where an insignificant decrease was observed, and plasminogen, where a statistically significant increase was observed between baseline and follow-up (see Figure 2). A detailed comparison of subgroups can be found in Table 1, and a detailed comparison of baseline and follow-up results can be found in Table 2.

### 3.2. Effect of LAA Orifice Size on Coagulation

A significant decrease in the analyzed coagulation factors between baseline and follow-up was observed at normal LAA orifice size for all parameters except plasminogen, for which a statistically nonsignificant decrease was observed. A significant decrease between baseline and follow-up was observed with enlarged LAA openings in all parameters except TAFI, in which the observed change was not statistically significant, and plasminogen, in which a statistically significant increase was observed between baseline and follow-up (see Figure 2). A detailed comparison of baseline and follow-up results can be found in Table 3, and a detailed comparison of subgroups can be found in Table 4.

## 4. Discussion

### 4.1. LAA Subtypes Impact on Postoperative Coagulation Changes

It is widely known that there is a relationship between the structure of the LA, disorders of the blood coagulation process and an increased risk of prothrombotic complications [7,14]. Our study fills the gap in understanding the relationship between hemostasis and LAA structure, including laboratory parameters assessing both blood coagulation and fibrinolysis. Our study provides detailed information about the influence of preoperative LAA anatomy on the changes in coagulation factors. It is the first study to discuss this topic in detail, connecting the molecular findings with anatomical parameters of cardiac structures. In the described population, it was observed that baseline plasminogen levels were higher in LAAs with small bodies than in LAAs with larger lobes. However, this was not demonstrated to be statistically significant. In contrast, there were significant differences between patients with large and small LAA orifices. Nevertheless, in both cases, mean plasminogen levels were stabilized at standard values at the 6-month coagulation factors analysis follow-up. The significant reduction in coagulation factor reduction differences in TAFI observed between the analyzed subgroups argues for potentially greater systemic coagulation changes favoring fibrinolysis in patients with smaller lobe dimensions and smaller orifices, which may play an additional role in preventing and reducing thromboembolic complications in patients in the future. There was no significant difference in the change in TAFI levels between baseline and follow-up in both large-lobe and large-orifice subgroups. This finding may be important in determining the potential negative systemic impact on fibrinolysis of the left atrial appendage and the closure of small left atrial appendages.

The intriguing relationship we observed was the lower levels of plasminogen in enlarged LAA orifices, which requires the study of this parameter in the context of the entire fibrinolytic system, including tissue plasminogen activator (tPA). This is extremely methodologically difficult due to the instability of this laboratory parameter in plasma. We cannot say conclusively that lower plasminogen levels in these patients increase the risk of thrombotic complications, but low plasminogen levels are associated with the severity of some diseases [25,26]. Low plasminogen levels may be associated with higher plasma PAI-1 levels, which are a well-established marker for major adverse cardiovascular events [27].

### 4.2. Does Left Atrial Appendage Type Matter?

This may suggest that orifice size, rather than LAA shape, may increase the risk of thromboembolism. Hypothetically, patients with a narrower LAA opening might experience increased turbulent blood flow and impaired blood outflow from the LAA, increasing the risk of thromboembolism. It may turn out that the size of the LAA appendage, rather than its shape, is crucial for the risk of thrombosis. However, this theory requires further extensive studies with a larger number of patients. It has already been demonstrated that AF promotes LAA remodeling, including dilation of the LAA orifice [28]. In this case, it could be an additional factor leading to thrombus formation within the LAA. The observed normalization of coagulation parameters over a 6-month period proves that the possible adverse effects can be reversed.

### 4.3. Molecular Findings Patophysiological Association

Our results are related to the two main causes of Virchow’s triad—stasis of blood flow and hypercoagulability in the remodeled, dilated LAA. The changes in fluid dynamics and geometry of the LAA, previously studied in detail, prove that the enlarged LAA further restricts blood flow through its lumen, which, combined with hypercoagulability in such patients, poses a major risk for thrombus formation, leading to catastrophic complications, including cryptogenic stroke and death. These results demonstrate the critical role of LAA occlusion procedures in the long-term prevention of thrombus formation in patients with AF, especially when DOAC or VKA cannot be administered because of the patient’s increased risk of bleeding.

The mechanism of clot formation after the LARIAT procedure is not fully understood, but considering the risk of this phenomenon being difficult to predict, it seems extremely important to look for the role of these phenomena in patient care [23]. In assessing such a risk, an in-depth analysis of the blood coagulation process seems important to more efficiently and quickly predict the risk of thrombosis after this type of surgery. The concept of assessing the viscoelastometric properties of the clot seems interesting here. Although these laboratory methods are largely associated with their usefulness during cardiac surgery, the evaluation of the clot elasticity of whole blood samples after the operation, e.g., using thromboelastometry, may bring surprising results in the evaluation of the post-LARIAT procedure.

### 4.4. Coagulation and Fibrynolitic Factors

Plasminogen is a plasma protein that can be activated by proteolysis in its active form—plasmin, which plays a crucial role in fibrinolysis. Its activity reference is 75% to 135%. It is worth noting that, in addition to its key fibrinolytic function, plasminogen also plays a crucial role in the behavior of cells in immune and inflammatory processes, including reducing the risk of infection and providing antiviral protection [29]. Fibrinogen plays a crucial role in the coagulation process, as it is the source of fibrin. Its reference range is 2–4 g/L. Its post-translational modifications have been shown to be associated with abnormal clot formation and thrombosis and can be found in patients with increased cardiovascular risk [25]. A thrombin-activatable fibrinolysis inhibitor is a plasma protein that plays an important role in protecting fibrin clots from degradation by removing C-terminal lysines from fibrin. It is critical for maintaining the balance between fibrinolysis and coagulation. It can be activated by several plasma proteins, including thrombin, in complex with thrombomodulin and plasmin. Elevated levels are associated with a hypofibrinolytic state, which increases the risk of thrombotic and cardiovascular disease [30]. Tissue plasminogen activator is a serine protease that converts plasminogen to plasmin, which is critical for blood clot dissolution. Its biological function is used in daily practice for the treatment of ischemic diseases such as stroke, pulmonary embolism, deep vein thrombolysis and in isolated cases of myocardial infarction [31]. Plasminogen activator inhibitor-1 plays a crucial role in the regulation and inhibition of tissue- and urinary-type plasminogen activators and blocks fibrinolysis at the stage of conversion of plasminogen to plasmin. It is produced in various cells and stored in platelets. Its increased expression in vivo can lead to pathological fibrin deposition and tissue damage. PAI-1 activity is increased by mental and psychological stress. Moreover, its increased activation has been found to be related to metabolic factors and the aging process [32].

### 4.5. Enlarged Left Atrial Appendage—How to Determine?

To define the enlarged LAA, one would first acknowledge its dimensions in a healthy population. In a healthy population analyzed by Veinot and colleagues, the mean longitudinal dimension of the LAA orifice was 1.16 ± 0.35 cm in males, with slightly lower values (was 1.07 ± 0.32 cm) in females. The length and width were 2.59 ± 0.66 cm and 1.83 ± 0.73 cm, respectively, with similar, slightly lower values in females [33]. The left atrial appendage can be divided into two main parts: body and neck. It was recently proven that the left atrial appendage neck, crucial in terms of blood flow and LAAO procedures, does not differ between the LAA morphological types [34].

The LAA enlargement was historically connected with rheumatic heart disease and evaluated subjectively [35,36]. Nowadays, there are still no clear criteria for defining the LAA enlargement. However, the study performed by Ren and colleagues proposed the first cutoff values for the left atrial appendage orifice diameter at the level of 23.6 mm [24]. In this study, we additionally propose the cutoff point of LAA body index at the level of 1000 mm^2^. There is still a great need for the evaluation and definition of cutoff values for each LAA parameter for a clear definition of its enlargement.

### 4.6. Clinical Impact of the Left Atrial Appendage Anatomy

LAA anatomy plays an important role in patient evaluation and qualification for specific LAAO procedures. Several LAA classifications have been proposed for easy evaluation, but we have not found significant differences between specific LAA types and clotting factor changes in follow-up [28,37]. LAA anatomy changes in patients with AF [28]. It was found that LAA orifice dimensions and lobe size differ between patients with AF and healthy individuals, with significantly larger values of the LAA orifice and significantly lower values of LAA ejection fraction observed in patients with AF, but there are limited data on the relationship between LA and LAA enlargement [28]. No data are available on how AF affects LAA size and dimensions in patients suffering from AF. To facilitate the implementation of the results in clinical practice, it was suggested that the LAA body index or orifice area should be determined, and patients divided into two subgroups. These measurements can be performed daily during the preoperative assessment of patients. As presented in our study, such data may provide additional information about potential postoperative coagulation factors in patients.

### 4.7. Endocardial Left Atrial Appendage Closure Coagulation Impact

Previously, several studies were conducted to determine endocardial LAAO and the impact of anticoagulation treatment on coagulation changes [38,39,40]. A study performed by Sherwood and colleagues aimed to determine the clinical characteristics of patients with one of the most commonly observed endocardial LAAO complications: device-related thrombus and postoperative bleeding [38]. It was proven that the prothrombogenic profile of the patients at baseline was associated with device-related thrombosis when low platelet reactivity was observed preoperatively in the bleeding group [38]. Asmarats and colleagues discovered that preoperative LAA thrombosis was observed only in patients with antiplatelet therapy and was associated with increased activation of coagulation factors postoperatively. In patients with oral anticoagulation treatment, such complications were not observed [39]. Additionally, Duthoit and colleagues compared rivaroxaban and dual antiplatelet therapy in patients undergoing endocardial LAAO, which proved that postoperative thrombin generation was lower in patients treated with lowered oral anticoagulation treatment doses, which proves it as an alternative to double antiplatelet therapy [40].

### 4.8. Limitations of the Study

This was a single-center prospective study performed only on patients of Caucasian origin. In addition, only 22 patients in whom epicardial LAAO was performed with the Lariat system were analyzed because data from other systems were not currently available. The blood analysis was performed before the procedure and at the 6-month follow-up. Additional analysis should be performed to establish long-term differences in patients’ coagulation factor changes after LAAO. One limitation of our study is the noticeable, although small, variability among patients in terms of drug treatment. It would be ideal to select a cohort without anticoagulant therapy, but in clinical practice, this is unlikely. However, it seems that the greatest impact on the tested coagulation and fibrinolysis parameters would be caused by vitamin K antagonists, which were used in only three patients in our current cohort. The proposed LAA body index should be clinically standardized and evaluated on a broad population of patients for standard value development. For the proposed hypothesis, an additional prospective study with a large number of patients divided into groups based on their LAA types and different LAA body and orifice sizes should be performed. This study was performed on patients with contraindications for oral anticoagulation, and its results should be interpreted in terms of such a population.

## 5. Conclusions

Closure of the left atrial appendage is beneficial with respect to systemic coagulation. Patients with a small LAA may benefit more from LAA closure in terms of stabilization of their coagulation factors associated with potential thromboembolic events in the future.

## Figures and Tables

**Table 1:** The left atrial appendage of normal and enlarged body subgroups detailed comparison. LAA—left atrial appendage, tPA—tissue plasminogen activator antigen, PAI-1—plasminogen activator inhibitor type 1 antigen, TAFI—thrombin activatable fibrinolysis inhibitor.

	Normal LAA Body	Enlarged LAA Body	*p*
	Mean ± SD	Mean ± SD	
LAA Width [mm]	16.7 ± 2.8	26.5 ± 6.9	<0.001
LAA Length [mm]	44.5 ± 10.9	49.6 ± 9.0	0.116
LAA body index [mm^2^]	727.1 ± 146	1324.5 ± 480.2	<0.001
Ostium transverse dimension [mm]	24.1 ± 5.2	32.1 ± 4.3	0.001
ostium longitudinal dimension [mm]	20.3 ± 4.3	25.8 ± 6.1	0.034
LAA ostium area [mm^2^]	398.3 ± 164.9	666.7 ± 240.0	0.010
LAA origin angle to LA [º]	64.8 ± 24.6	75.2 ± 9.5	0.748

**Table 2:** Baseline versus 6-month follow-up left atrial appendage normal and enlarged body subgroups detailed comparison. LAA—left atrial appendage, tPA—tissue plasminogen activator antigen, PAI-1—plasminogen activator inhibitor type 1 antigen, TAFI—thrombin activatable fibrinolysis inhibitor.

	Normal LAA Body			Enlarged LAA Body		
	Baseline (Mean ± SD)	6-Month Follow-Up (Mean ± SD)	Baseline vs. 6-Month Follow-Up *p*	Baseline (Mean ± SD)	6-Month Follow-Up (Mean ± SD)	Baseline vs. 6-Month Follow-Up *p*
Plasminogen (%)	109.1 ± 30	97.7 ± 14.6	0.181	89.5 ± 12.0	105.2 ± 14.6	0.007
Fibrinogen g/L	4.4 ± 0.9	2.9 ± 0.8	0.020	3.9 ± 0.9	2.6 ± 0.7	0.003
tPA conc. (ng/mL)	10.6 ± 3.6	5.8 ± 2.1	0.003	10.5 ± 6.8	6.8 ± 4.9	0.007
PAI-1 (ng/mL)	3.8 ± 2.6	2.3 ± 1.5	0.003	4.9 ± 3.1	2.3 ± 1.5	0.007
TAFI (%)	101.1 ± 12.2	82.7 ± 12.5	0.003	105.2 ± 19.6	96 ± 17.6	0.108

**Table 3:** The left atrial appendage of normal and enlarged orifice subgroups detailed comparison. LAA—left atrial appendage, tPA—tissue plasminogen activator antigen, PAI-1—plasminogen activator inhibitor type 1 antigen, TAFI—thrombin activatable fibrinolysis inhibitor.

	Normal LAA Orifice	Enlarged LAA Orifice	*p*
	Mean ± SD	Mean ± SD	
LAA Width [mm]	19.1 ± 5.6	24.2 ± 7.9	0.116
LAA Length [mm]	43.1 ± 11.1	51.0 ± 7.6	0.034
LAA body index [mm^2^]	791.6 ± 207.5	1260 ± 533.3	0.016
Ostium transverse dimension [mm]	23.7 ± 4.6	32.6 ± 4.2	<0.001
ostium longitudinal dimension [mm]	18.2 ± 1.6	27.8 ± 4.4	<0.001
LAA ostium area [mm^2^]	344.0 ± 93.8	720.9 ± 193.7	<0.001
LAA origin angle to LA [º]	66.9 ± 26.3	73.1 ± 6.7	0.300

**Table 4:** Baseline versus 6-month follow-up left atrial appendage of normal and enlarged orifice subgroups detailed comparison. LAA—Left atrial appendage, tPA—tissue plasminogen activator antigen, PAI-1—plasminogen activator inhibitor type 1 antigen, TAFI—thrombin activatable fibrinolysis inhibitor.

	Normal LAA Orifice			Enlarged LAA Orifice		
	Baseline (Mean ± SD)	6-Month Follow-Up (Mean ± SD)	Baseline vs. 6-Month Follow-Up *p*	Baseline (Mean ± SD)	6-Month Follow-Up (Mean ± SD)	Baseline vs. 6-Month Follow-Up *p*
Plasminogen (%)	111.9 ± 28.3	103.1 ± 9.9	0.422	86.7 ± 10.3	99.8 ± 18.9	0.007
Fibrinogen g/L	4.2 ± 0.9	2.7 ± 0.8	0.003	4.0 ± 0.8	2.8 ± 0.8	0.020
tPA conc. (ng/mL)	9.4 ± 1.3	5.6 ± 1.1	0.004	11.6 ± 7.4	7 ± 5.2	0.006
PAI-1 (ng/mL)	4.5 ± 3.4	2.2 ± 1.6	0.007	4.2 ± 2.3	2.4 ± 1.4	0.003
TAFI (%)	104.2 ± 13.4	80.2 ± 11.5	0.003	102 ± 19.0	98.4 ± 15.7	0.284

## Data Availability

The data from the study are available upon reasonable request from the corresponding author.

## Appendix A

**Table A1:** Patients’ baseline demographic and clinical characteristics. BMI—body mass index, OAC—oral anticoagulant, SD—standard deviation, TIA—transient ischemic attack.

Variable	Patients (*n* = 22)
Age, years (Mean ± SD)	66.2 ± 8.7
Female	59.1% (*n* = 13)
BMI (Mean ± SD) [kg/m^2^]	26.4 ± 2.4
CHADS_2_ score (Mean ± SD)	2.8 ± 1.0
CHA_2_DS_2_-VASc score (Mean ± SD)	3.9 ± 1.5
HAS-BLED score (Mean ± SD)	3.6 ± 1.2
Congestive heart failure	13.6% (*n* = 3)
Hypertension	86.4% (*n* = 19)
Hyperlipidemia	54.5% (*n* = 12)
Diabetes mellitus type 2	31.8% (*n* = 7)
Previous ischemic stroke/TIA	68.2% (*n* = 15)
Hemorrhagic stroke	59.1% (*n* = 13)
Myocardial infarction	9.1% (*n* = 2)
Vascular disease	13.6% (*n* = 3)
Alcoholism	4.5% (*n* = 1)
Preprocedural OAC	
- Vitamin K antagonist - Direct oral anticoagulant - Aspirin - None	13.6% (*n* = 3) 50.0% (*n* = 11) 13.6% (*n* = 3) 22.7% (*n* = 5)

**Table A2:** Anatomical characteristics of the left atrial appendage. LA—left atrium, LAA—left atrial appendage.

		*n* (%)/Mean ± SD
LAA type	chicken wing	9 (40.9%)
	cauliflower	2 (9.1%)
	arrowhead	11 (50%)
LAA additional lobes	0	11 (50%)
	1	9 (40.9%)
	2	2 (9.1%)
Orientation of main lobe	superior anterolateral	13 (59.1%)
	superior posterolateral	5 (22.7%)
	superior lateral	4 (18.2%)
LAA width [mm]	21.6 ± 7.2	
LAA length [mm]	47.1 ± 10.1	
LAA body index [mm^2^]	1025.8 ± 462.0	
Ostium transverse dimension [mm]	28.1 ± 6.2	
Ostium longitudinal dimension [mm]	23 ± 5.9	
LAA ostium area [mm^2^]	532.5 ± 243.4	
LAA origin angle to LA [º]	70.0 ± 19.0	
